# Efficacy comparison of PD-1/PD-L1 inhibitor monotherapy and combination with PARPis or antiangiogenic agents in advanced or recurrent endometrial cancer: a systematic review and network meta-analysis

**DOI:** 10.1186/s12905-025-03612-7

**Published:** 2025-02-28

**Authors:** Shiya Ji, Xupeng Chen, Yebo Yu, Qiuping Jia, Xingxing Zhang, Zixin Gao

**Affiliations:** 1 Department of Health Education, Nanjing Municipal Center for Disease Control and Prevention, No.16 Kunlun Road, Nanjing, 210003 Jiangsu Province China; 2 Department of Social Medicine and Health Education, School of Public Health, Peking University, Beijing, China; 3 Department of Health Education, Jiangning District Center for Disease Control and Prevention, Nanjing, China; 4 High School Affiliated to Nanjing Normal University, Nanjing, China

**Keywords:** Network meta-analysis, Endometrial cancer, Immunotherapy, Programmed cell death protein 1, Programmed cell death ligand 1

## Abstract

**Purpose:**

The network meta-analysis (NMA) was aimed to compare and assess the effectiveness of programmed cell death 1 (PD-1)/ programmed cell death ligand 1 (PD-L1) inhibitor monotherapy or combination therapy with other agents for individuals with advanced or recurrent endometrial cancer (EC).

**Methods:**

The NMA was registered on the PROSPERO website (ID: CRD42024545968) and multiple databases were queried to retrieve the articles. It assessed the progression-free survival (PFS) and overall survival (OS) of persons with advanced or recurrent EC, as well as those with deficient mismatch repair (dMMR) and proficient mismatch repair (pMMR) in terms of PFS.

**Results:**

The NMA included 12 studies involving a total of 4,515 patients. Compared to chemotherapy, the PD-1/PD-L1 inhibitor monotherapy (hazard ratio [HR], 0.59; 95% confidence interval [CI]: 0.44–0.78) in PFS, combination therapy with poly (ADP-ribose) polymerase inhibitors (PARPis) (HR, 0.53; 95% CI: 0.32–0.89) or with antiangiogenic agents (HR, 0.48; 95% CI: 0.25–0.83) all showed significant improvements in PFS. PD-1/PD-L1 inhibitor monotherapy resulted in a significantly higher OS (HR, 0.61; 95% CI: 0.37–0.97) compared to chemotherapy. Combination therapy with antiangiogenic agents demonstrated the highest efficacy in extending PFS, while the combination with PARPis had the best performance in extending OS. Patients with dMMR and pMMR subtypes derive greater benefits from PD-1/ PD-L1 inhibitor monotherapy and PD-1/PD-L1 inhibitors combined with PARPis respectively.

**Conclusion:**

Monotherapy with PD-1/PD-L1 inhibitors and combination therapies with PARPis or antiangiogenic agents demonstrate significant potential for individuals with advanced or recurrent EC.

**Supplementary Information:**

The online version contains supplementary material available at 10.1186/s12905-025-03612-7.

## Introduction

Uterine corpus carcinoma is the sixth most common cancer in women globally, with 420,242 new cases in 2022, representing 4.3% of all new cancer diagnoses in women [1]. Its incidence and death rates are on the rise worldwide [2]. Endometrial cancer (EC) is the predominant subtype of uterine corpus cancer and represents approximately 83% of cases [3]. Although early-stage EC generally has a favorable prognosis, the five-year survival rate for individuals diagnosed with advanced or recurrent EC remains dismally low, ranging between 20% and 25% [4]. Currently, the accepted approach for treating advanced or recurrent EC is the administration of cytotoxic chemotherapeutic drugs, such as carboplatin and paclitaxel. This treatment yields a response rate of approximately 10-15%, which is considered favorable [5]. Innovative and effective treatment strategies are critically needed for individuals with advanced or recurrent EC who face a poor prognosis.

The Cancer Genome Atlas Research Project verified four genomic subtypes of EC, among them polymerase epsilon mutations and deficient DNA mismatch repair (dMMR) / microsatellite instability-high (MSI-H) have elevated mutation rates, resulting in increased neoantigens and penetration of CD8 + T-cells [6]. The immune response activates tumor immune resistance, characterized by the upregulation of anti-programmed cell death 1 (PD-1) and anti-programmed cell death ligand 1 (PD-L1) [7]. Studies [8–13] suggested that immune checkpoint inhibitors (ICIs) targeting PD-1/PD-L1 exhibit promising in managing EC, especially in those exhibiting dMMR/MSI-H. Nevertheless, the impact remains questionable in patients with proficient mismatch repair (pMMR) [12, 13]. Recent clinical trials have investigated combination approaches to uncover synergistic effects with ICIs, hence boosting clinical efficacy. These explorations have primarily concentrated on the combination of ICIs with antiangiogenic agents or poly(ADP-ribose) polymerase inhibitors (PARPis) [4]. The phase III trial KEYNOTE-775 [14] found that lenvatinib plus pembrolizumab contributed to significant enhancements in progression-free survival (PFS) and overall survival (OS) for both the overall population as well as the pMMR subgroups. Similarly, the DUO-E study [8] reported that durvalumab plus olaparib significantly prolonged OS and PFS in the complete participant group, and improved PFS in the pMMR subgroup.

Although PD-1/PD-L1 inhibitors have revealed effectiveness in treating advanced or recurrent EC, either as monotherapy or in combination, the superiority of combination therapy over monotherapy remains uncertain. This is particularly true when considering the added expenses and potential toxicities associated with combination therapy. This study utilized a network meta-analysis (NMA) to evaluate and compare the effectiveness of PD-1/PD-L1 inhibitor-based therapies for advanced or recurrent EC. The evaluated treatments included PD-1/PD-L1 inhibitor monotherapy, PD-1/PD-L1 inhibitors plus PARPis, and PD-1/PD-L1 inhibitors plus antiangiogenic agents. The examination also included clinical efficacy across subgroups of dMMR and pMMR.

## Methods

The NMA was performed using the standards set forth by the Cochrane Collaboration’s Preferred Reporting Items for Systematic Reviews and Meta-Analyses (PRISMA) [15]. The protocol was officially documented on the PROSPERO website with a unique registration ID: CRD42024545968. All procedures were conducted in complete adherence to the registered protocol.

### Search strategy

Two independent researchers did a thorough search across databases such as PubMed, Web of Science, Cochrane library, and Embase to find relevant papers. No restrictions were placed on the publication dates, but only English-language publications were included. Additionally, references from included studies and prior reviews were meticulously analyzed to ensure no relevant research was overlooked. The search phrases primarily consisted of terms like endometrial carcinoma, immune checkpoint inhibitors, and PD-1/PD-L1 inhibitors. The methodology for retrieving literature was described in Table S1 (Supplementary Material). We utilized EndNote X9 to combine the search results and eliminate duplicates.

### Study selection

Trials were considered eligible when meeting the specified inclusion criteria: (1) diagnosed advanced or recurrent EC patients who underwent treatment with PD-1/PD-L1 inhibitor either alone or combined with PARPis or antiangiogenic agents; (2) patients were compared to a control group, which could consist of standard chemotherapy or other treatments; (3) studies that reported PFS and/or OS; (4) study designs that included cohort studies, case-control studies, clinical trials of randomized controlled trials (RCTs) or non-RCTs. The criteria for disqualification encompassed the following: (1) studies with less than 10 included patients; (2) reviews or comments; (3) laboratory research conducted on animals or cells; (4) detailed accounts of individual cases; and (5) trials where all participants receive the same treatment.

### Data extraction

Two reviewers, S JI and X Chen, conducted a comprehensive analysis of relevant studies and carefully documented the data in electronic spreadsheets. The recorded material contained essential information, such as author’s name, age, year of publication, number of participants, molecular type (dMMR or pMMR), and efficacy endpoints of PFS and/or OS. OS is defined as the duration from the moment of random assignment to the occurrence of death. PFS is defined as the duration between the random assignment and the occurrence of disease progression or death, whichever happened first. Two independent reviewers evaluated the risk of bias with the ROB2 (Risk of Bias 2) for RCTs [16], while the non-RCTs were evaluated using the Newcastle-Ottawa Scale [17]. When using the ROB2 tool to assess the risk of bias, the evaluation criteria are divided into three levels based on the specific circumstances of each domain: Low risk, Some concerns, and High risk. Low risk of bias is assigned when a trial demonstrates low risk across all domains. Some concerns are noted if there is a concern in at least one domain, but none are classified as high risk. High risk of bias is determined when at least one domain is at high risk, or there are concerns in multiple domains that substantially reduce confidence in the result [18]. Any conflicts or discrepancies were resolved by consulting with a third independent reviewer, Y Yu.

### Statistical analysis

This NMA was conducted by R software (version 4.3.2) and the “gemtc” package. We used the hazard ratio (HR) and a 95% confidence interval (CI) in the random-effect model to assess the impact on survival outcomes of PFS and OS. Subgroup analyses were performed based on the EC molecular subtypes of dMMR and pMMR to evaluate PFS. HR values below 1 for PFS and OS were considered as indicative of favorable outcomes. A Markov chain Monte Carlo simulation was employed to create pooled HR, with a burn-in of 20,000 and 50,000 iterations. The trace plot, density plot, and Gelman-Rubin plot were applied to evaluate the convergence of the model. The node split methodology was employed to evaluate the discrepancy between direct and indirect evidence.

All following analyses were conducted for both entire population and subgroups. Forest plots were conducted to display direct and indirect comparisons among various regimes. Pairwise comparisons were generated by synthesizing studies that compared the same interventions into a random effects model. We also applied surface under the cumulative ranking (SUCRA) technique to evaluate the efficacy of the treatment interventions offered. Higher SUCRA scores in the same comparison group indicate better efficacy of agents. I² statistics were applied to assess heterogeneity, with I² value above 50% denoting substantial heterogeneity. Sensitivity analysis was conducted for further analysis when the heterogeneity between the included studies was high or when missing data were present. Statistical significance was defined as a two-tailed *p*-value below 0.05 or a 95% CI for the pooled HR that did not include 1.

## Results

### Study characteristics

**Fig. 1:**
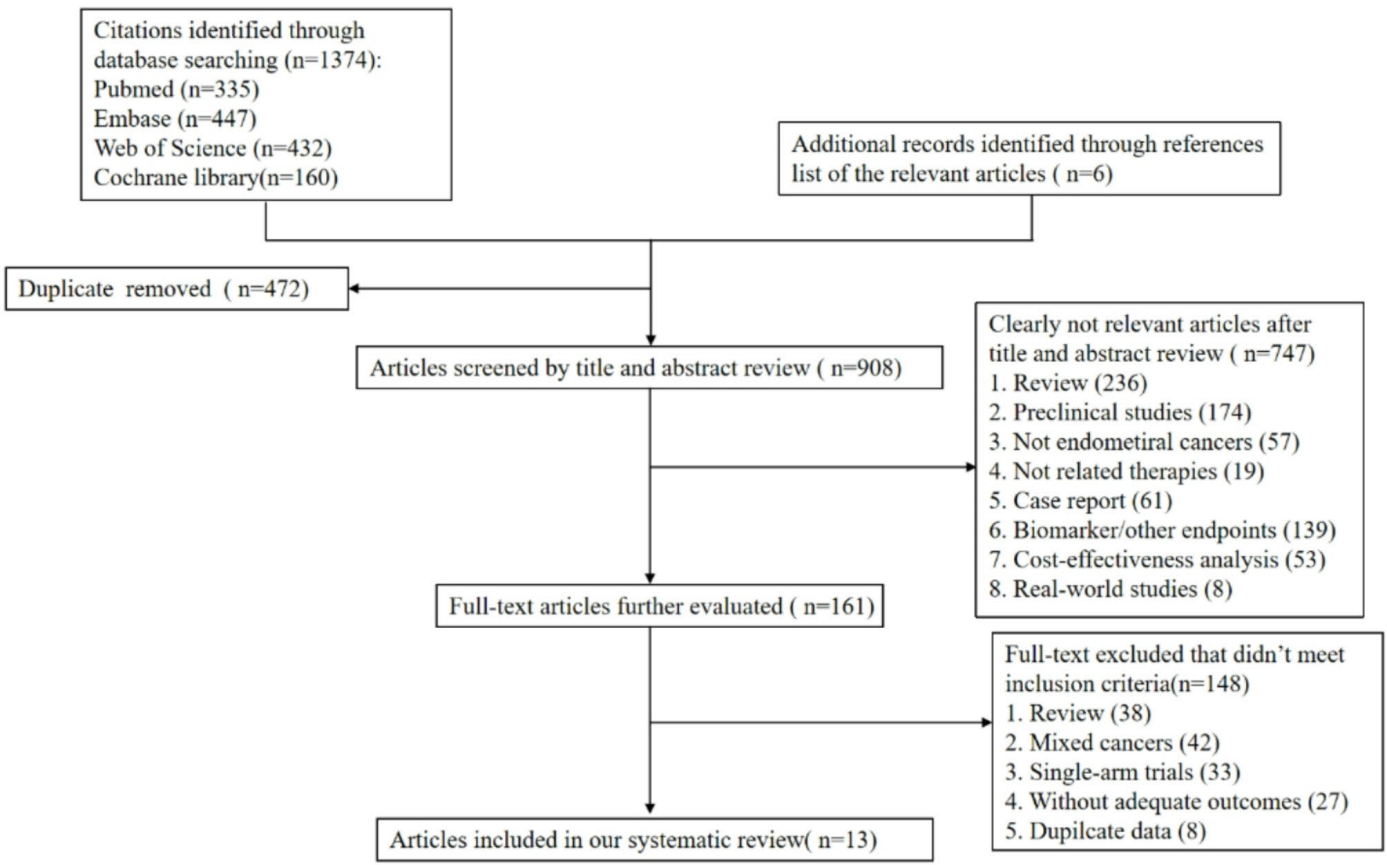
Study flow diagram

**Fig. 2:**
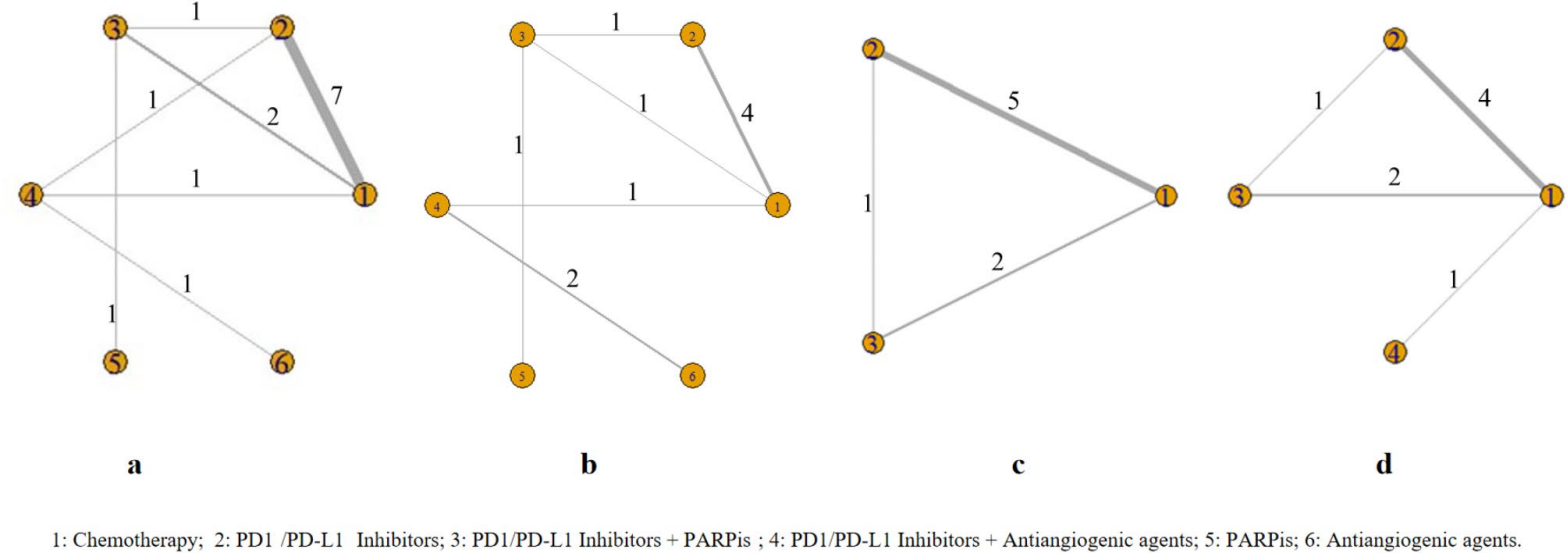
Network plots for each of the different outcomes assessed (**a**) progression-free survival, (**b**) overall survival, (**c**) progression-free survival of deficient mismatch repair subgroups, (**d**) progression-free survival of proficient mismatch repair subgroups. Each node refers to a certain type of treatment: (1) Chemotherapy; (2) PD11/PD-L12 Inhibitors; (3) PD1/PD-L1 Inhibitors + PARPis; (4) PD1/PD-L1 Inhibitors + Antiangiogenic agents; (5) PARPis; (6) Antiangiogenic agents. The width of the lines is proportional to the number of comparisons (beside the line) comparing the connected treatment (nodes). A total of 14 comparisons were analyzed for progression-free survival (**a**), a total of 10 comparisons were analyzed for overall survival (**b**), a total of 8 comparisons were analyzed for (**c**) progression-free survival of deficient mismatch repair subgroups, a total of 8 comparisons were analyzed for progression-free survival of proficient mismatch repair subgroups (**d**)

The NMA discovered a total of 1,380 publications that were potentially relevant. After removing 472 duplicate publications and 747 items that did not match the eligibility conditions, a comprehensive evaluation was conducted on a total of 161 publications for full-text examination. Ultimately, the NMA included 13 publications, which cover 12 trials conducted from 2022 to 2024 [5, 8–11, 13, 14, 19–24]. The summary characteristics of the included studies are presented in Table 1. Figure 1 illustrates the flow of the literature search. These studies encompassed 4,396 patients across six treatment groups: chemotherapy alone, PD-1/PD-L1 inhibitor monotherapy, PD-1/PD-L1 inhibitors plus PARPis, PD-1/PD-L1 inhibitors plus antiangiogenic agents, PARPi monotherapy, and antiangiogenic agent monotherapy. Figure 2 displays the network plots, illustrating the direct comparisons of PFS and OS. Each node in Fig. 2 refers to a certain type of treatment, covering 6 regimes. The width of the lines is proportional to the number of comparisons (beside the line) comparing the connected treatment (nodes). A total of 14 comparisons were analyzed for PFS and a total of 10 comparisons were analyzed for OS. 8 comparisons were analyzed for both dMMR and pMMR in PFS. The majority of RCTs included in this NMA were assessed as low risk of bias, while non-RCTs were of moderate to high quality (Table S2 and S3). All of the incorporated RCTs were published in prestigious medical publications known for their excellent quality.

**Table 1:** Characteristics of the included studies

Author Year	Treatment	Sample size	Follow-up (months)	Age (years)	Population	PFS^4^ (entire) HR(95%CI)	PFS (dMMR) HR(95%CI)	PFS (pMMR) HR(95%CI)	OS^5^(entire) HR(95%CI)	Outcomes
Westin et al.[8] 2023 (DUO-E)	CP^1^ + durvalumab	238	15.4	64 (22–84)	entire dMMR^2^ pMMR^3^	0.71(0.57–0.89)	0.42(0.22–0.89)	0.77(0.6–0.97)	0.77(0.56–1.07)	PFS OS
	CP + durvalumab + olaprib	239	15.4	63(27–86)		0.55(0.43–0.69)	0.41(0.21–0.75)	0.57(0.44–0.73)	0.59(0.42–0.83)	
	CP	241	12.6	64 (22–84)		Ref	Ref	Ref	Ref	
Eskander et al.[9] 2023 (NRG-GY018)	CP + pembrolizumab	112	12	67(38–81)	dMMR	-	0.3(0.19–0.48)	-	-	PFS
	CP + placebo	113		67 (38–81)	dMMR		Ref			
	CP + pembrolizumab	293	7.9	67 (38–81)	pMMR		-	0.54(0.41–0.71)		
	CP + placebo	295		67 (38–81)	pMMR			Ref		
Makker et al.[10, 14] 2022 (KEYNOTE-775)	lenvatinib + pembrolizumab	411	12.2	64 (30–82)	entire	0.56(0.48–0.66)		0.6(0.5–0.72)	0.65(0.55–0.77)	PFS OS
	doxorubicin/ paclitaxel	416	10.7	65 (35–86)		Ref		Ref	Ref	
Mirza et al.[11] 2023 (RUBY part1)	CP + dostarlimab	245	25.4	64 (41–81)	entire dMMR	0.64(0.51–0.8)	0.28(0.16–0.5)	0.76(0.59–0.98)	0.64(0.46–0.87)	PFS OS
	CP + placebo	249		65 (28–85)		Ref	Ref	Ref	Ref	
Mirza et al. [22] 2024 (RUBY part2)	CP + dostarlimab + niraparib	192	19	-	dMMR	0.6(0.43–0.82)	0.48(0.24–0.96)	0.63(0.44–0.91)	-	PFS
	CP + placebo	99		-		Ref	Ref	Ref		
Colombo et al.[23] 2023 (AtTEnd)	CP + atezolizumab	360	28.3	67 (61–73)-	entire	0.74(0.61–0.91)	0.36(0.23–0.57)	-	-	PFS
	CP + placebo	189		65 (60–73)		Ref	Ref			
Pignata et al.[13] 2023 (MITO END-3)	CP + avelumab	62	23.3	65 (56–70)	entire dMMR pMMR	0.78(0.51–1.19)	0.46(0.22–0.94)	1.17(0.65-0.1)	1.13(0.62–2.07)	PFS OS
	CP	62		66 (61–72)		Ref	Ref	Ref	Ref	
Lheureux et al.[24] 2022	nivolumab + cabozantinib	39	15.9	67 (44–77)	entire	0.54(0.27–1.06)	-	-	-	PFS
	nivolumab	20		66 (41–83)		Ref				
Madariaga et al.[19] 2023	niraparib	25	-	69 (53–80)	entire	Ref	-	-	1.4(0.54–3.67)	PFS OS
	niraparib + dostarlimab	22		64.5(38–80)		0.87(0.49–1.55)			Ref	
Mathews et al.[5] 2022	dostarlimab	92	-	63.3	entire	0.38(0.28–0.51)	-	-	0.41(0.28–0.61)	PFS OS
	doxorubicin	233		63.7		Ref			Ref	
Liao et al.[20] 2022	nivolumab + bevacizumab	52	-	46.71 ± 6.42	entire	-	-	-	0.35(0.19–0.65)	OS
	bevacizumab	41		45.22 ± 5.27					Ref	
Cui et al.[21] 2022	anlotinib	44	13.5	62 (42–80)	entire	1.83(0.47–7.11)	-	-	0.84(0.26–2.80)	PFS OS
	anlotinib + pembrolizumab	12				Ref			Ref	

^1^carboplatin+paclitaxel; ^2^deficient mismatch repair;^3^proficient mismatch repair;^4^progression-free survival; ^5^ overall survival

### PFS

**Fig. 3:**
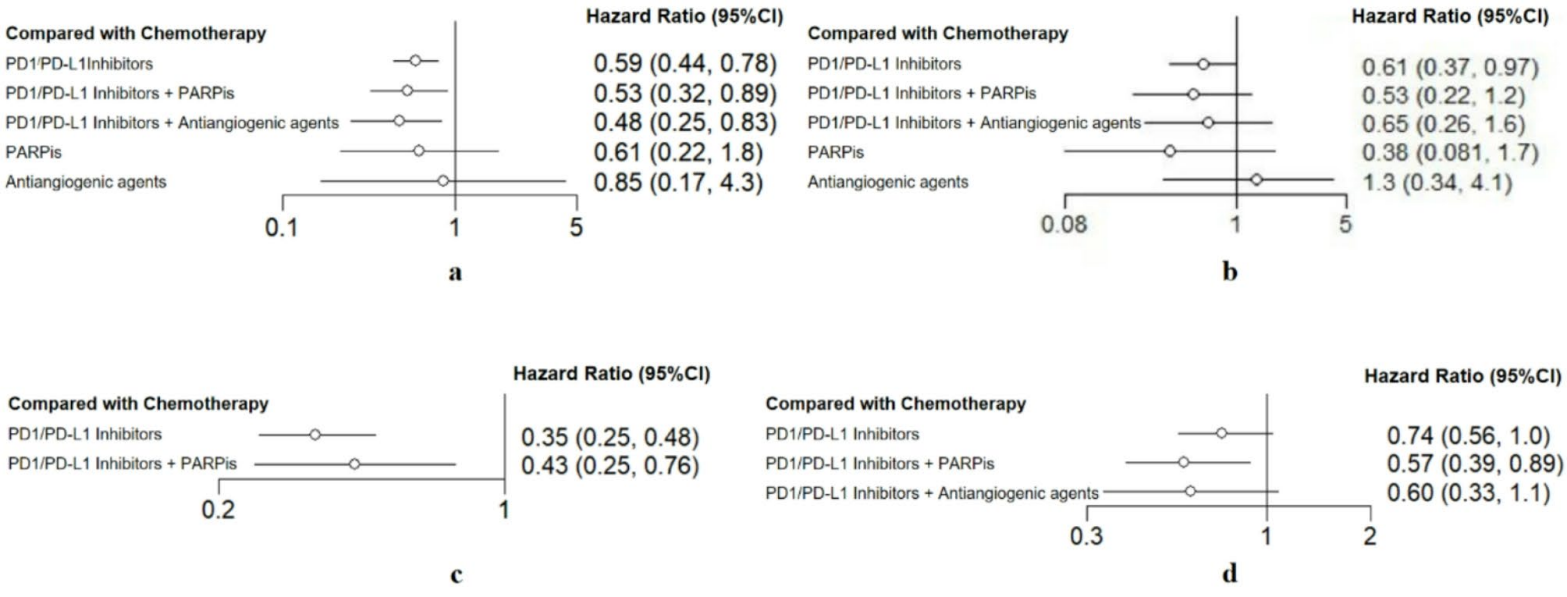
Forest plots representing the effect of treatment on (**a**) progression-free survival, (**b**) overall survival, (**c**) progression-free survival of deficient mismatch repair subgroups, (**d**) progression-free survival of proficient mismatch repair subgroups

The NMA included 10 studies reporting 13 PFS outcomes. The DUO-E study [8] included three treatment arms and separately reported the PFS for PD-1/PD-L1 inhibitor monotherapy, combined with antiangiogenic agents, and chemotherapy alone, with each treatment analyzed separately. The RUBY trial [11] compared PD-1/PD-L1 inhibitor monotherapy versus chemotherapy, and PD-1/PD-L1 inhibitors plus PARPis versus chemotherapy. The NRG-GY018 study provided separate PFS outcomes for dMMR and pMMR cohorts. Minimal heterogeneity was observed among the studies, as indicated by an I² value of 15%. PD-1/PD-L1 inhibitor alone revealed a notable improvement in PFS (HR, 0.59; 95% CI: 0.44–0.78) in comparison to chemotherapy. Similarly, combination therapy with PARPis (HR, 0.53; 95% CI: 0.32–0.89) and with antiangiogenic agents (HR, 0.48; 95% CI: 0.25–0.83) substantially prolonged PFS. However, neither PARPi alone (HR, 0.61; 95% CI: 0.22–1.75) nor antiangiogenic agent alone (HR, 0.87; 95% CI: 0.17–4.37) showed significant advantages over chemotherapy. Forest plots for all treatment comparisons are displayed in Fig. 3, while pairwise comparisons for the overall population are displayed in Table 2. Among all regimes, PD-1/PD-L1 inhibitors plus antiangiogenic agents had the highest efficacy in PFS with a SUCRA value of 0.814. Detailed SUCRA values for all therapies and outcomes are presented in Table 3.

**Table 2:** Network meta-analysis of pairwise comparisons for progression-free survival (PFS) and overall survival (OS) of entire population across various treatment regimens

**Pairwise Comparisons for PFS of Entire Population (upper triangle)**					
Chemotherapy	**0.59 (0.44**,** 0.78)**	**0.53 (0.32**,** 0.89)**	**0.48 (0.25**,** 0.83)**	0.61 (0.22, 1.75)	0.87 (0.17, 4.37)
**0.61 (0.37**,** 0.97)**	PD1/PD-L1 Inhibitors	0.89 (0.54, 1.55)	0.8 (0.42, 1.43)	1.03 (0.37, 3.01)	1.48 (0.28, 7.49)
0.53 (0.22, 1.24)	0.87 (0.36, 2.05)	PD1/PD-L1 Inhibitors + PARPis	0.9 (0.39, 1.83)	1.15 (0.46, 2.85)	1.64 (0.29, 8.73)
0.65 (0.26, 1.64)	1.07 (0.38, 3.06)	1.24 (0.35, 4.44)	PD1/PD-L1 Inhibitors + Antiangiogenic agents	1.29 (0.41, 4.47)	1.85 (0.41, 8.4)
0.38 (0.08, 1.72)	0.62 (0.13, 2.85)	0.72 (0.2, 2.56)	0.58 (0.1, 3.39)	PARPis	1.43 (0.2, 9.51)
1.33 (0.34, 4.09)	2.18 (0.52, 7.42)	2.52 (0.5, 10.24)	2.01 (0.79, 4.38)	3.48 (0.45, 22.81)	Antiangiogenic agents
**Pairwise Comparisons for OS of Entire Population (lower triangle)**					

The upper triangle presents hazard ratios (HRs) with 95% confidence intervals (CIs) for PFS, while the lower triangle displays HRs with 95% CIs for OS. Bolded values indicate statistically significant results (95% CI does not cross 1). For upper triangle, an HR < 1 favors the column-defining treatment, while an HR > 1 favors the row-defining treatment. For lower triangle, an HR < 1 favors the row-defining treatment, while an HR > 1 favors the column-defining treatment. Treatments include chemotherapy, PD-1/PD-L1 inhibitors, and their combinations with PARP inhibitors (PARPis) or antiangiogenic agents. This table facilitates direct comparisons between treatment strategies, providing insights into relative efficacy in terms of survival outcomes

**Table 3:** Rank probabilities with surface under the cumulative ranking curve (SUCRA) value for different outcomes for advanced or recurrent endometrial patients

Treatment	PFS^1^ of Entire Population	PFS of dMMR^2^ Population	PFS of pMMR^3^ Population	OS^4^
Chemotherapy	0.268	0.335	0.270	0.330
PD1/PD-L1 Inhibitors	0.620	0.924	0.562	0.671
PD1/PD-L1 Inhibitors + PARPis	0.730	0.741	0.867	0.755
PD1/PD-L1 Inhibitors + Antiangiogenic agents	0.814	-	0.800	0.630
PARPis	0.610	-	-	0.841
Antiangiogenic agents	0.458	-	-	0.272

^1^progression-free suvival; ^2^ deficient mismatch repair; ^3^ proficient mismatch repair; ^4^ overall survival

The higher scores indicate more favorable outcomes

The analyses of the dMMR and pMMR subgroups included six trials reporting seven PFS outcomes, with heterogeneity measures (I²) of 0% and 23%, respectively. In the dMMR population, PD-1/PD-L1 inhibitor monotherapy substantially prolonged PFS (HR, 0.35; 95% CI: 0.25–0.48), as did the combination of PD-1/PD-L1 inhibitors and PARPis (HR, 0.43; 95% CI: 0.25–0.76). Notably, PD-1/PD-L1 inhibitor alone achieved the highest SUCRA score (0.924) for PFS in this subgroup. In the pMMR population, PD-1/PD-L1 inhibitor combined with PARPis had a notable enhancement in PFS (HR, 0.57; 95% CI: 0.39–0.89) and ranked highest (SUCRA, 0.867) compared to other therapies. Neither PD-1/PD-L1 inhibitor alone nor combined with antiangiogenic agents showed significant benefits over chemotherapy in the pMMR subgroup. Subgroup head-to-head comparisons are presented in Table 4.

**Table 4:** Network meta-analysis of pairwise comparisons for progression-free survival (PFS) of pMMR population and dMMR population across various treatment regimens

**Pairwise Comparisons for PFS of pMMR** ^**1**^ **Population (upper triangle)**			
Chemotherapy	**0.74 (0.56**,** 1.03)**	**0.57 (0.39**,** 0.89)**	**0.6 (0.33**,** 1.08)**
**0.35 (0.25**,** 0.48)**	PD1/PD-L1 Inhibitors	0.78 (0.49, 1.21)	0.81 (0.41, 1.54)
**0.43 (0.25**,** 0.76)**	1.25 (0.66, 2.33)	PD1/PD-L1 Inhibitors + PARPis	1.05 (0.5, 2.09)
NA	NA	NA	PD1/PD-L1 Inhibitors + Antiangiogenic agents
**Pairwise Comparisons for PFS of dMMR** ^**2**^ **Population (lower triangle)**			

^**1**^proficient mismatch repair; ^**2**^deficient mismatch repair

The upper triangle presents hazard ratios (HRs) with 95% confidence intervals (CIs) for PFS of pMMR Population, while the lower triangle displays HRs with 95% CIs for PFS of dMMR Population. Bolded values indicate statistically significant results (95% CI does not cross 1). For upper triangle, an HR < 1 favors the column-defining treatment, while an HR > 1 favors the row-defining treatment. For lower triangle, an HR < 1 favors the row-defining treatment, while an HR > 1 favors the column-defining treatment. Treatments include chemotherapy, PD-1/PD-L1 inhibitors, and their combinations with PARP inhibitors (PARPis) or antiangiogenic agents. This table facilitates direct comparisons between treatment strategies, providing insights into relative efficacy in terms of survival outcomes.

### OS

The NMA included 8 trials reporting 9 OS outcomes. The DUO-E study [8] separately reported OS for PD-1/PD-L1 inhibitor alone, PD-1/PD-L1 inhibitors plus antiangiogenic agents, and chemotherapy. The heterogeneity level was moderate, with an I² value of 22%. Of the therapies evaluated, only PD-1/PD-L1 inhibitor monotherapy notably enhanced OS (HR, 0.61; 95% CI: 0.37–0.97) in comparison to chemotherapy. No significant OS benefits were observed for PD-1/PD-L1 inhibitors plus PARPis (HR, 0.53; 95% CI: 0.22–1.24), PD-1/PD-L1 inhibitor plus antiangiogenic agents (HR, 0.65; 95% CI: 0.26–1.64), PARPis monotherapy (HR, 0.38; 95% CI: 0.08–1.72), or antiangiogenic agent monotherapy (HR, 1.33; 95% CI: 0.34–4.09) compared to chemotherapy. PARPi monotherapy achieved the highest SUCRA score (0.841) for OS.

## Disccussion

Treatment options for advanced or recurrent EC have remained largely unchanged over recent decades [4]. The response rate to conventional chemotherapy in these individuals ranges from 10 to 15% [4], and the five-year survival rate for advanced-stage patients is between 20% and 25% [5], highlighting a critical need for more effective therapies. The emergence of ICIs is rapidly changing this grim scenario. Recent meta-analyses [25–28] have indicated that PD-1/PD-L1 inhibitors outperform conventional regimes in improving overall response rate, PFS, and OS in advanced or recurrent EC patients. However, the quantitative assessment of combination therapies and the comparative evaluation of different treatment regimens remain uncertain. Our NMA demonstrated that PD-1/PD-L1 inhibitors, whether utilized as single-agent therapy or combination treatment, considerably prolonged PFS compared to chemotherapy alone. Among all regimens, PD-1/PD-L1 inhibitors plus antiangiogenic agents demonstrated the highest efficacy in PFS, followed by PD-1/PD-L1 inhibitors plus PARPis and PD-1/PD-L1 inhibitor monotherapy. Both PD-1/PD-L1 inhibitor used as single-therapy and combined with PARPis markedly extended PFS for the dMMR subgroups, while only the combination therapy with PARPis substantially enhanced PFS for the pMMR subgroups. PD-1/PD-L1 inhibitor monotherapy was the only treatment that demonstrated a substantial benefit in terms of OS. Among all regimens, PARPi monotherapy achieved the highest SUCRA ranking for OS, followed by PD-1/PD-L1 inhibitors plus PARPis, PD-1/PD-L1 monotherapy, and PD-1/PD-L1 inhibitors plus antiangiogenic agents.

Based on emerging clinical trial evidence, the 2022 ESMO Clinical Practice Guidelines [29] provided key recommendations of anti-PD-1/PD-L1 agents, including pembrolizumab, avelumab, durvalumab, dostarlimab, atezolizumab, and nivolumab, for the treatment of advanced or recurrent MSI-H or dMMR endometrial tumors, as well as those with high tumor mutational burden. The KEYNOTE-775 study demonstrated that pembrolizumab combined with lenvatinib significantly improved overall survival compared to doxorubicin or paclitaxel monotherapy in patients with advanced endometrial cancer who had prior standard treatment. Based on the results from the NRG-GY018 and RUBY trials, the 2025 NCCN Guidelines for Endometrial Carcinoma recommend pembrolizumab/carboplatin/paclitaxel and dostarlimab/carboplatin/paclitaxel as Category 1, preferred first-line therapies for recurrent endometrial carcinoma [30].

Reports [31–33] indicate that approximately 17–36% of EC patients have dMMR tumors, which are often associated with advanced, high-grade disease and lymphovascular invasion. Despite receiving comparable treatment, advanced stage (III/IV) EC patients with dMMR exhibit higher recurrence rates (47.7% vs. 3.4%) compared to those with pMMR [33]. The dMMR subgroup analysis indicated that both PD-1/PD-L1 inhibitor used as single-agent therapy and combination with PARPis significantly prolonged PFS, while PD-1/PD-L1 inhibitor alone achieved higher efficacy. It suggests that adding PARPis to PD-1/PD-L1 inhibitors may not bring extra benefit within dMMR subgroup, underscoring the importance of molecular diagnostics for treatment stratification. The significance of immunotherapy for patients with pMMR tumors is also critical. Our study found that only PD-1/PD-L1 inhibitors plus PARPis provided significant enhancement in PFS within the pMMR subgroup. However, most included trials demonstrated significant benefits in PFS compared to chemotherapy, such as PD-1/PD-L1 monotherapy (DUO-E, NRG-GY018, and RUBY) or combinations with antiangiogenic agents (KEYNOTE-775). The failure of the avelumab group in the MITO-END3 study to reach median PFS could potentially affect the overall assessment of treatment efficacy in the pMMR subgroup analysis.

Two studies significantly influenced the PARPi-related OS outcomes. The DUO-E study [8] reported that duvalumab plus olaparib significantly prolonged OS (HR, 0.59; 95% CI:0.42–0.83) compared to chemotherapy. Conversely, Madariaga et al. [19] showed no significant OS improvement with niraparib plus dostarlimab (HR, 1.4; 95% CI: 0.54–3.67) compared to niraparib monotherapy. The median OS for the combination group was not determined at the time of data assessment, which may obscure its true benefit compared to monotherapy. Consequently, the real effectiveness of PD-1/PD-L1 inhibitors plus PARPis in prolonging OS may be underestimated, while the ranking of PARPi monotherapy could be overestimated.

Preclinical studies [34, 35] suggest that PD-1/PD-L1 inhibitors combined with other agents produces synergistic effects through unique mechanisms. PARPis enhance the expression of neoantigens and promote immune recognition by inducing DNA damage, which complements the immune checkpoint blockade via PD-1/PD-L1 inhibition [24]. This synergy is facilitated by the activation of the stimulator of interferon genes (STING) pathway [25]. Additionally, antiangiogenic agents have been shown to reduce immune-suppressive cells, such as T regulatory cells and myeloid-derived suppressor cells, while increasing CD4 + and CD8 + T-cell populations and reducing PD-1 expression, thereby enhancing the immune response [36–38].

Immunotherapy has shown potential in fertility-sparing treatments for endometrial cancer, especially in young patients. A case reported by Cao et al. [39] highlighted a 36-year-old patient with Lynch syndrome-associated synchronous endometrial and colon cancer who was treated with sintilimab, a PD-1 inhibitor. Despite initial pelvic lymph node metastases, her reproductive organs were preserved after treatment, and she successfully conceived and delivered a healthy baby. This suggests that immunotherapy may offer fertility preservation options for select patients. The anti–PD-1 toliparimab, in combination with standard megestrol acetate, will be evaluated as a fertility-sparing treatment for patients with stage I endometrioid grade 1 or 2 endometrial cancer who wish to preserve fertility (NCT04046185) [40]. However, careful consideration is needed regarding the use of immunotherapy during pregnancy due to potential impacts on fetal development, as animal studies indicated that PD-1/PD-L1 blockade can disrupt maternal-fetal immune tolerance [41]. Additionally, the suitability of fertility-sparing treatments depends on factors like myometrial invasion, FIGO staging, and molecular classification [42]. Tumors with specific molecular subtypes, such as POLE mutations, and biomarkers like L1CAM or CTNNB1 mutations, may be more amenable to fertility preservation. A multidisciplinary approach involving gynecologists, oncologists, and fertility specialists is crucial to optimize treatment and fertility preservation.

Balancing the benefits and risks of cancer treatment is crucial for extending survival, controlling symptoms, and improving quality of life. Immune checkpoint inhibitors can cause immune dysregulation and also typical chemotherapy toxicities [43]. A meta-analysis by Nishijima et al. [44] found that PD-1/PD-L1 inhibitors are generally better tolerated than chemotherapy. PD1/PD-L1 inhibitors were associated with a lower risk of treatment‐related symptoms and hematologic toxicities. Most of the immune‐related AEs were low-grade, but high‐grade events were described, especially pneumonitis [45]. 68% of endometrial cancer patients receiving PD-1/PD-L1 inhibitor monotherapy reported experiencing AEs related to the treatment [28]. Grade 3 or higher adverse events were observed in 15% of patients. But grade 1 or 2 AEs typically did not result in significant clinical issues for patients. These findings corroborate the work of Zhao et al. [46], which suggests that the motivation of receiving PD-1/PD-L1 inhibitors is generally not influenced by the occurrence of side effects.

Additionally, it is essential to assess the adverse events related combination therapy, which were not added to our NMA because of the limited number of studies. Research has reported that the adverse reactions of PD-1/PD-L1 inhibitors are primarily related to immune system activation [47]. According to Maiorano et al. [47], PD-1/PD-L1 inhibitors were linked to an increased occurrence of rash, pruritus, and thyroid function abnormalities compared to chemotherapy. However, they also exhibited a more favorable safety profile in reducing fatigue, gastrointestinal issues, hematologic toxicity, and treatment discontinuations in solid tumors [44]. According to Han et al. [26], the most common adverse events of PD-1/PD-L1 inhibitors in EC patients were hypertension, anemia, exhaustion, and severe skin reactions. The DUO-E study [8] reported that the occurrence of grade 3 or above treatment emergent adverse events (TEAEs) was 56.4%, 54.9%, and 67.2% in the control group, durvalumab monotherapy, and durvalumab combined with olaparib groups respectively. The KEYNOTE-775 trial [14] revealed that 90.1% of patients administered lenvatinib plus pembrolizumab and 73.7% of those given chemotherapy encountered grade 3 or more severe TEAEs. These findings suggest that combination therapy poses significant risks, therefore, safety monitoring is essential in clinical practice.

### Implications

Our NMA further reinforces the efficacy of PD-1/PD-L1 inhibitor as single-agent therapy and combination therapies in the treatment of advanced or recurrent EC, offering insights for clinical practice. PD-1/PD-L1 inhibitors plus antiangiogenic agents demonstrate the greatest PFS benefit in managing advanced or recurrent EC. However, patients with pMMR and dMMR achieve the most PFS benefit from PD-1/PD-L1 inhibitors combined PARPis and PD-1/PD-L1 inhibitor monotherapy respectively, underscoring the critical role of molecular diagnostics in guiding personalized strategies. PD1/PD-L1 inhibitors plus PARPis ranked highest in prolonging OS, while only PD1/PD-L1inhibitor monotherapy demonstrated a significant OS benefit. It suggests that long-term follow-up data are urgently required to measure the overall survival outcomes associated with different therapies. Moreover, clinicians must closely manage immune-related adverse effects, particularly with combination therapies. Future studies should also further investigate the mechanisms underlying synergy in combination therapies.

### Limitations

Firstly, the articles obtained by this NMA may have disregarded certain papers published in languages other than English due to linguistic limitations, which could introduce publication bias. Additionally, there were no restrictions on the number of previous treatment regimens for EC patients, which could influence the consistency of the results. Furthermore, the available OS data for EC patients was insufficient, necessitating a more comprehensive follow-up to accurately determine a more stabilized value of overall survival. The RUBY part 2 study included in this NMA has only been published as an abstract, so the data on patient age are unavailable. Madariaga et al. [19], Mathews et al. [5] and Liao et al. [20] did not report the follow-up duration in their published article, thus the follow-up time is missing in these studies. Finally, safety data was not subjected to quantitative analysis because the included studies reported adverse occurrences from diverse perspectives.

## Conclusion

Administering PD-1/PD-L1 inhibitor, either as single-agent or combined with PARPis or antiangiogenic agents, has significantly prolonged PFS for persons with advanced or recurrent EC. PD-1/PD-L1 inhibitor monotherapy and combined treatment with PARPis resulted in a notable enhancement of PFS in subgroups with dMMR. In contrast, PFS in patients with pMMR subtypes was significantly extended only when PD-1/PD-L1 inhibitors were combined with PARPis. Notably, only PD-1/PD-L1 inhibitor alone demonstrated a significant improvement in OS.

## Electronic supplementary material

Below is the link to the electronic supplementary material.

Supplementary Material 1

Supplementary Material 2

## Data Availability

All data relevant to the study are included in the article or uploaded as supplementary information.

## References

[1] Bray F, Laversanne M, Sung H, Ferlay J, Siegel RL, Soerjomataram I et al. Global cancer statistics 2022: GLOBOCAN estimates of incidence and mortality worldwide for 36 cancers in 185 countries. 2024;74(3):229–63. 10.3322/caac.21834 38572751

[2] Siegel RL, Miller KD, Wagle NS, Jemal A, Cancer statistics. 2023. CA: a cancer journal for clinicians. 2023;73(1):17–48. 10.3322/caac.21763 36633525

[3] Mahdy H, Casey MJ, Vadakekut ES, Crotzer D. Endometrial Cancer. StatPearls. Treasure Island (FL) ineligible companies. StatPearls Publishing LLC.; 2024. 30252237

[4] Grau-Bejar JF, Farinas-Madrid L, García-Duran C, García-Illescas D, Mazzeo R, Oaknin AJCAH, et al. Immunotherapy Treat Adv or Recurr Endometrial Cancer. 2024;22(3):129–39. 38588272

[5] Mathews C, Lorusso D, Coleman RL, Boklage S, Garside J. An Indirect comparison of the efficacy and safety of Dostarlimab and Doxorubicin for the treatment of Advanced and recurrent endometrial Cancer. Oncologist. 2022;27(12):1058–66. 36124638 10.1093/oncolo/oyac188 PMC9732237

[6] Kandoth C, Schultz N, Cherniack AD, Akbani R, Liu Y, Shen H, et al. Integrated genomic characterization of endometrial carcinoma. Nature. 2013;497(7447):67–73. 23636398 10.1038/nature12113 PMC3704730

[7] Howitt BE, Shukla SA, Sholl LM, Ritterhouse LL, Watkins JC, Rodig S, et al. Association of polymerase e-Mutated and microsatellite-instable endometrial cancers with Neoantigen load, number of Tumor-infiltrating lymphocytes, and expression of PD-1 and PD-L1. JAMA Oncol. 2015;1(9):1319–23. 26181000 10.1001/jamaoncol.2015.2151

[8] Westin SN, Moore K, Chon HS, Lee JY, Thomes Pepin J, Sundborg M, et al. Durvalumab Plus Carboplatin/Paclitaxel followed by maintenance Durvalumab with or without Olaparib as First-Line treatment for Advanced Endometrial Cancer: the Phase III DUO-E trial. J Clin Oncology: Official J Am Soc Clin Oncol. 2024;42(3):283–99. 10.1200/JCO.23.02132 PMC10824389 37864337

[9] Eskander RN, Sill MW, Beffa L, Moore RG, Hope JM, Musa FB, et al. Pembrolizumab plus Chemotherapy in Advanced Endometrial Cancer. N Engl J Med. 2023;388(23):2159–70. 36972022 10.1056/NEJMoa2302312 PMC10351614

[10] Makker V, Colombo N, Casado Herráez A, Santin AD, Colomba E, Miller DS, et al. Lenvatinib plus Pembrolizumab for Advanced Endometrial Cancer. N Engl J Med. 2022;386(5):437–48. 35045221 10.1056/NEJMoa2108330 PMC11651366

[11] Mirza MR, Chase DM, Slomovitz BM, dePont Christensen R, Novák Z, Black D, et al. Dostarlimab for Primary Advanced or recurrent endometrial Cancer. N Engl J Med. 2023;388(23):2145–58. 36972026 10.1056/NEJMoa2216334

[12] Colombo N, Harano K, Hudson E, Galli F, Antill Y, Choi CH, et al. LBA40 phase III double-blind randomized placebo controlled trial of atezolizumab in combination with carboplatin and paclitaxel in women with advanced/recurrent endometrial carcinoma. Ann Oncol. 2023;34:S1281–2. 10.3802/jgo.2025.36.e117 PMC12226327 40590326

[13] Pignata S, Scambia G, Schettino C, Arenare L, Pisano C, Lombardi D, et al. Carboplatin and paclitaxel plus avelumab compared with carboplatin and paclitaxel in advanced or recurrent endometrial cancer (MITO END-3): a multicentre, open-label, randomised, controlled, phase 2 trial. Lancet Oncol. 2023;24(3):286–96. 37052965 10.1016/S1470-2045(23)00016-5

[14] Makker V, Colombo N, Casado Herráez A, Monk BJ, Mackay H, Santin AD, et al. Lenvatinib Plus Pembrolizumab in previously treated Advanced Endometrial Cancer: updated efficacy and safety from the Randomized Phase III Study 309/KEYNOTE-775. J Clin Oncology: Official J Am Soc Clin Oncol. 2023;41(16):2904–10. 10.1200/JCO.22.02152 PMC10414727 37058687

[15] Page MJ, McKenzie JE, Bossuyt PM, Boutron I, Hoffmann TC, Mulrow CD et al. The PRISMA 2020 statement: an updated guideline for reporting systematic reviews. 2021;372. 10.1136/bmj.n71 PMC8005924 33782057

[16] Sterne JA, Savović J, Page MJ, Elbers RG, Blencowe NS, Boutron I et al. RoB 2: a revised tool for assessing risk of bias in randomised trials. 2019;366. 10.1136/bmj.l4898 31462531

[17] Wells GA, Shea B, O’Connell D, Peterson J, Welch V, Losos M et al. The Newcastle-Ottawa Scale (NOS) for assessing the quality of nonrandomised studies in meta-analyses. 2000.

[18] Higgins JP, Savović J, Page MJ, Elbers RG. Sterne JAJChfsroi. Assessing risk of bias in a randomized trial. 2019:205– 28.

[19] Madariaga A, Garg S, Tchrakian N, Dhani NC, Jimenez W, Welch S, et al. Clinical outcome and biomarker assessments of a multi-centre phase II trial assessing niraparib with or without dostarlimab in recurrent endometrial carcinoma. Nat Commun. 2023;14(1):1452. 36922497 10.1038/s41467-023-37084-w PMC10017680

[20] Liao Y, Zhu C, Song X, Ruan J, Ding Y, Chen Y, et al. Efficacy of PD-1 inhibitor combined with Bevacizumab in Treatment of Advanced Endometrial Cancer patients with Mismatch Repair Deficiency (dMMR)/High-Level microsatellite instability (MSI-H). Med Sci Monitor: Int Med J Experimental Clin Res. 2022;28:e934493. 10.12659/MSM.934493 PMC8962632 35322001

[21] Cui Q, Mao Y, Hu Y, Ma D, Liu H. Anlotinib in recurrent or metastatic endometrial cancer. Int J Gynecol cancer: Official J Int Gynecol Cancer Soc. 2022;32(9):1147-1152. 10.1136/ijgc-2022-003345 35606048

[22] Mirza M, Ghamande S, Hanker L, Black D, Raascou-Jensen N, Gilbert L et al. Dostarlimab plus chemotherapy followed by dostarlimab plus niraparib maintenance therapy among patients with primary advanced or recurrent endometrial cancer in the ENGOT-EN6-NSGO/GOG-3031/RUBY trial. 2024;190:S6.

[23] Colombo N, Biagioli E, Harano K, Galli F, Hudson E, Antill Y et al. Atezolizumab and chemotherapy for advanced or recurrent endometrial cancer (AtTEnd): a randomised, double-blind, placebo-controlled, phase 3 trial. 2024;25(9):1135–46. 10.1016/S1470-2045(24)00334-6 39102832

[24] Lheureux S, Matei DE, Konstantinopoulos PA, Wang BX, Gadalla R, Block MS et al. Translational randomized phase II trial of cabozantinib in combination with nivolumab in advanced, recurrent, or metastatic endometrial cancer. J Immunother Cancer. 2022;10(3). 10.1136/jitc-2021-004233 PMC8921950 35288469

[25] Bartoletti M, Montico M, Lorusso D, Mazzeo R, Oaknin A, Musacchio L, et al. Incorporation of anti-PD1 or anti PD-L1 agents to platinum-based chemotherapy for the primary treatment of advanced or recurrent endometrial cancer. A meta-analysis. Cancer Treat Rev. 2024;125:102701. 38422895 10.1016/j.ctrv.2024.102701

[26] Han S, Guo C, Song Z, Ouyang L, Wang Y. Effectiveness and safety of PD-1/PD-L1 inhibitors in advanced or recurrent endometrial cancer: a systematic review and meta-analysis. Front Pharmacol. 2023;14:1330877. 38161705 10.3389/fphar.2023.1330877 PMC10755929

[27] Kim JH, Han KH, Park EY, Kim ET, Kim EJ, Tan DSP, et al. Efficacy of immune-checkpoint inhibitors combined with cytotoxic chemotherapy in advanced or recurrent endometrial cancer: a systematic review and meta-analysis. Gynecol Oncol. 2024;187:85–91. 38735144 10.1016/j.ygyno.2024.05.006

[28] Wan X, Huang J, Huang L, Wang Y, Fu Y, Jin X, et al. Effectiveness and safety of PD-1/PD-L1 inhibitors monotherapy in patients with endometrial cancer. Discov Oncol. 2024;15(1):168. 38750182 10.1007/s12672-024-01033-w PMC11096149

[29] Oaknin A, Bosse TJ, Creutzberg CL, Giornelli G, Harter P, Joly F, et al. Endometrial cancer: ESMO Clinical Practice Guideline for diagnosis, treatment and follow-up. Ann Oncol. 2022;33(9):860–77. 35690222 10.1016/j.annonc.2022.05.009

[30] Network NCC. NCCN Clinical Practice Guidelines in Oncology-Uterine Neoplasms(Version 1.2025). 2024.

[31] McMeekin DS, Tritchler DL, Cohn DE, Mutch DG, Lankes HA, Geller MA et al. Clinicopathologic significance of mismatch repair defects in endometrial cancer: an NRG oncology/gynecologic oncology group study. 2016;34(25):3062. 10.1200/JCO.2016.67.8722 PMC5012715 27325856

[32] Aghajanian C, Filiaci V, Dizon DS, Carlson JW, Powell MA, Secord AA et al. A phase II study of frontline paclitaxel/carboplatin/bevacizumab, paclitaxel/carboplatin/temsirolimus, or ixabepilone/carboplatin/bevacizumab in advanced/recurrent endometrial cancer. 2018;150(2):274–81. 10.1016/j.ygyno.2018.05.018 PMC6179372 29804638

[33] Cosgrove CM, Cohn DE, Hampel H, Frankel WL, Jones D, McElroy JP et al. Epigenetic silencing of MLH1 in endometrial cancers is associated with larger tumor volume, increased rate of lymph node positivity and reduced recurrence-free survival. 2017;146(3):588–95. 10.1016/j.ygyno.2017.07.003 PMC5601318 28709704

[34] Post CCB, Westermann AM, Bosse T, Creutzberg CL, Kroep JR. PARP and PD-1/PD-L1 checkpoint inhibition in recurrent or metastatic endometrial cancer. Crit Rev Oncol/Hematol. 2020;152:102973. 32497971 10.1016/j.critrevonc.2020.102973

[35] Shen J, Zhao W, Ju Z, Wang L, Peng Y, Labrie M et al. PARPi triggers the STING-dependent immune response and enhances the therapeutic efficacy of immune checkpoint blockade independent of BRCAness. 2019;79(2):311–9. 10.1158/0008-5472.CAN-18-1003 PMC6588002 30482774

[36] Ozao-Choy J, Ma G, Kao J, Wang GX, Meseck M, Sung M et al. The novel role of tyrosine kinase inhibitor in the reversal of immune suppression and modulation of tumor microenvironment for immune-based cancer therapies. 2009;69(6):2514–22. 10.1158/0008-5472.CAN-08-4709 PMC4370269 19276342

[37] Kwilas AR, Ardiani A, Donahue RN, Aftab DT, Hodge, JWJJotm. Dual effects of a targeted small-molecule inhibitor (cabozantinib) on immune-mediated killing of tumor cells and immune tumor microenvironment permissiveness when combined with a cancer vaccine. 2014;12:1–15. 10.1186/s12967-014-0294-y PMC4236498 25388653

[38] Kwilas AR, Donahue RN, Tsang KY. Hodge JWJCc, microenvironment. Immune consequences of tyrosine kinase inhibitors that synergize with cancer immunotherapy. 2015;2(1). 10.14800/ccm.677 PMC4440700 26005708

[39] Cao D, Gao Y, Zhang R-x, Wang F-l, Li C, Wu M-q et al. Case report: Reproductive organ preservation and subsequent pregnancy for an infertility patient with lynch syndrome-associated synchronous endometrial cancer and colon cancer after treatment with a PD-1 checkpoint inhibitor. 2022;13. 10.3389/fimmu.2022.1010490 PMC9618861 36325347

[40] Marín-Jiménez JA, García-Mulero S, Matías-Guiu X, Piulats JM. Facts and hopes in Immunotherapy of Endometrial Cancer. Clin cancer Research: Official J Am Association Cancer Res. 2022;28(22):4849–60. 10.1158/1078-0432.CCR-21-1564 35789264

[41] Poulet FM, Wolf JJ, Herzyk DJ, DeGeorge JJJBDRPBD, Toxicology R. An evaluation of the impact of PD-1 pathway blockade on reproductive safety of therapeutic PD‐1 inhibitors. 2016;107(2):108–19. 10.1002/bdrb.21176 27062127

[42] Dellino M, Cerbone M, Laganà AS, Vitagliano A, Vimercati A, Marinaccio M, et al. Upgrading treatment and molecular diagnosis in Endometrial Cancer—Driving. New Tools Endometrial Preservation? 2023;24(11):9780. 10.3390/ijms24119780 PMC10253366 37298731

[43] Michot J, Bigenwald C, Champiat S, Collins M, Carbonnel F, Postel-Vinay S et al. Immune-related adverse events with immune checkpoint blockade: a comprehensive review. 2016;54:139–48. 10.1016/j.ejca.2015.11.016 26765102

[44] Nishijima TF, Shachar SS, Nyrop KA, Muss HBJT. Safety and tolerability of PD-1/PD‐L1 inhibitors compared with chemotherapy in patients with advanced cancer: a meta‐analysis. 2017;22(4):470–9. 10.1634/theoncologist.2016-0419 PMC5388381 28275115

[45] Naidoo J, Page D, Li BT, Connell LC, Schindler K, Lacouture ME et al. Toxicities of the anti-PD-1 and anti-PD-L1 immune checkpoint antibodies. 2015;26(12):2375–91. 10.1093/annonc/mdv383 PMC6267867 26371282

[46] Zhao X, Gao F, Yang J, Fan H, Xie Q, Jiang K et al. Risk of adverse events in cancer patients receiving nivolumab with ipilimumab: a meta-analysis. 2022;12:877434. 10.3389/fonc.2022.877434 PMC9260026 35814436

[47] Boutros C, Tarhini A, Routier E, Lambotte O, Ladurie FL, Carbonnel F et al. Safety profiles of anti-CTLA-4 and anti-PD-1 antibodies alone and in combination. 2016;13(8):473–86. 10.1038/nrclinonc.2016.58 27141885

