# Supplementary material for: Efficacy comparison of PD-1/PD-L1 inhibitor monotherapy and combination with PARPis or antiangiogenic agents in advanced or recurrent endometrial cancer: a systematic review and network meta-analysis
Source: BMC Womens Health. 2025 Feb 28;25:93. doi: 10.1186/s12905-025-03612-7 (PMC11869547; doi:10.1186/s12905-025-03612-7)
Supplement: Supplementary file 1 — Supplementary Material 1 [file 12905_2025_3612_MOESM1_ESM.docx]

**Supplementary Material**

**Efficacy Comparison of PD-1/PD-L1 inhibitor monotherapy and Combination with PARPis or Antiangiogenic Agents in Advanced or Recurrent Endometrial Cancer: A Systematic Review and Network Meta-analysis**

Shiya Ji^1†^*, Xupeng Chen^1†^, Yebo Yu^2^, Qiuping Jia^3^, Xingxing Zhang^3^, Zixin Gao^4^,

1 Department of Health Education, Nanjing Municipal Center for Disease Control and Prevention, Nanjing, China

2 Department of Social Medicine and Health Education, School of Public Health, Peking University, Beijing, China

3 Department of Health Education, Jiangning District Center for Disease Control and Prevention, Nanjing, China.

4 High School Affiliated to Nanjing Normal University, Nanjing, China.

† These authors have contributed equally to this work

^*^Corresponding author:

Shiya Ji,

Department of Health Education, Nanjing Municipal Center for Disease Control and Prevention, No.16 Kunlun Road, 210003, Nanjing, Jiangsu Province of CHINA

E-mail: [jishiya@126.com](mailto:jishiya@126.com)

**List of abbreviations**

| PD-1 | = | Anti-programmed cell death 1 |
| --- | --- | --- |
| PD-L1 | = | Anti-programmed cell death ligand 1 |
| PARPis | = | poly (ADP-ribose) polymerase inhibitors |
| OS | = | Overall Survival |
| PFS | = | Progression-free Survival |
| pMMR | = | proficient mismatch repair |
| dMMR | = | deficient mismatch repair |

**Table S1.** Electronic search strategies

| **Pubmed** |
| --- |
| 1. "Endometrial Neoplasms"[Mesh] 2. "Endometrial Neoplasms"[Title/Abstract] OR "Endometrial Neoplasm"[Title/Abstract] OR "Neoplasm, Endometrial"[Title/Abstract] OR "Neoplasms, Endometrial"[Title/Abstract] OR "Endometrial Carcinoma"[Title/Abstract] OR "Carcinoma, Endometrial"[Title/Abstract] OR "Carcinomas, Endometrial"[Title/Abstract] OR "Endometrial Carcinomas"[Title/Abstract] OR "Endometrial Cancer"[Title/Abstract] OR "Cancer, Endometrial"[Title/Abstract] OR "Cancers, Endometrial"[Title/Abstract] OR "Endometrial Cancers"[Title/Abstract] OR "Endometrium Cancer"[Title/Abstract] OR "Cancer, Endometrium"[Title/Abstract] OR "Cancers, Endometrium"[Title/Abstract] OR "Cancer of the Endometrium"[Title/Abstract] OR "Carcinoma of Endometrium"[Title/Abstract] OR "Endometrium Carcinoma"[Title/Abstract] OR "Endometrium Carcinomas"[Title/Abstract] OR "Cancer of Endometrium"[Title/Abstract] OR "Endometrium Cancers"[Title/Abstract] 3. #1 or #2 4. "Immune Checkpoint Inhibitors"[Mesh] 5. "Immune Checkpoint Inhibitors"[Title/Abstract] OR "Checkpoint Inhibitors, Immune"[Title/Abstract] OR "Immune Checkpoint Inhibitor"[Title/Abstract] OR "Checkpoint Inhibitor, Immune"[Title/Abstract] OR "Immune Checkpoint Blockers"[Title/Abstract] OR "Checkpoint Blockers, Immune"[Title/Abstract] OR "Immune Checkpoint Blockade"[Title/Abstract] OR "Checkpoint Blockade, Immune"[Title/Abstract] OR "Immune Checkpoint Inhibition"[Title/Abstract] OR "Checkpoint Inhibition, Immune"[Title/Abstract] OR "PD-L1 Inhibitors"[Title/Abstract] OR "PD L1 Inhibitors"[Title/Abstract] OR "PD-L1 Inhibitor"[Title/Abstract] OR "PD L1 Inhibitor"[Title/Abstract] OR "Programmed Death-Ligand 1 Inhibitors"[Title/Abstract] OR "Programmed Death Ligand 1 Inhibitors"[Title/Abstract] OR "PD-1-PD-L1 Blockade"[Title/Abstract] OR "Blockade, PD-1-PD-L1"or "PD 1 PD L1 Blockade"[Title/Abstract] OR "PD-1 Inhibitors"[Title/Abstract] OR "PD 1 Inhibitors"[Title/Abstract] OR "PD-1 Inhibitor"[Title/Abstract] OR "Inhibitor, PD-1"or "PD 1 Inhibitor"[Title/Abstract] OR "Programmed Cell Death Protein 1 Inhibitor"[Title/Abstract] OR "Programmed Cell Death Protein 1 Inhibitors"[Title/Abstract] 6. "Durvalumab"[Title/Abstract] OR "MEDI4736"[Title/Abstract] OR "MEDI-4736"[Title/Abstract] OR "Imfinzi"[Title/Abstract] OR "Pembrolizumab"[Title/Abstract] OR "SCH-900475"[Title/Abstract] OR "lambrolizumab"[Title/Abstract] OR "MK-3475"[Title/Abstract] OR "Keytruda"[Title/Abstract] OR "Dostarlimab"[Title/Abstract] OR "Jemperli"[Title/Abstract] OR "dostarlimab-gxly"[Title/Abstract] OR "TSR-042"[Title/Abstract] OR "GSK4057190"[Title/Abstract] OR "Atezolizumab"[Title/Abstract] OR "anti-PDL1"[Title/Abstract] OR "immunoglobulin G1, anti-(human CD antigen CD274) (human monoclonal MDPL3280a heavy chain), disulfide with human monoclonal MDPL3280a kappa-chain, dimer"[Title/Abstract] OR "MPDL3280A"[Title/Abstract] OR "MPDL-3280A"[Title/Abstract] OR "Tecentriq"[Title/Abstract] OR "RG7446"[Title/Abstract] OR "RG-7446"[Title/Abstract] OR "Nivolumab"[Title/Abstract] OR "Opdivo"[Title/Abstract] OR "ONO-4538"[Title/Abstract] OR "ONO 4538"[Title/Abstract] OR "ONO4538"[Title/Abstract] OR "MDX-1106"[Title/Abstract] OR "MDX 1106"[Title/Abstract] OR "MDX1106"[Title/Abstract] OR "BMS-936558"[Title/Abstract] OR "BMS 936558"[Title/Abstract] OR "BMS936558"[Title/Abstract] OR "Avelumab"[Title/Abstract] OR "MSB-0010682"[Title/Abstract] OR "MSB0010682"[Title/Abstract] OR "bavencio"[Title/Abstract] OR "MSB0010718C"[Title/Abstract] OR "MSB-0010718C"[Title/Abstract] 7. #4 or #5 or #6 8. #3 and #7 9. ("randomized controlled trial"[Publication Type] OR "controlled clinical trial"[Publication Type] OR "randomized"[Title/Abstract] OR "placebo"[Title/Abstract] OR "drug therapy"[Mesh Subheading] OR "randomly"[Title/Abstract] OR "trial"[Title/Abstract] OR "groups"[Title/Abstract]) NOT ("animals"[MeSH Terms] NOT "humans"[MeSH Terms]) or "clinical trials as topic"[mesh] OR "random allocation"[mesh] OR "double-blind method"[mesh] OR "single-blind method"[mesh] OR "clinical trial"[pt] OR "research design"[mesh:noexp] OR "comparative study"[pt] OR "evaluation studies"[pt] OR "follow-up studies"[mesh] OR "prospective studies"[mesh] OR "cross-over studies"[mesh] OR "clinical trial"[tw] OR ((singl*[tw] OR doubl*[tw] OR trebl*[tw]) AND (mask*[tw] OR blind*[tw])) OR placebo*[tw] OR random*[tw] OR "control"[tw] OR "controls"[tw] OR prospectiv*[tw] OR volunteer*[tw] or "cohort studies"[mesh] OR "case-control studies"[mesh] OR "comparative study"[pt] OR "risk factors"[mesh] OR "cohort"[tw] OR "compared"[tw] OR "groups"[tw] OR "case control"[tw] OR "multivariate"[tw] 10. #8 or #9 |
| **Web of Science** |
| 1. TS=(“Endometrial Neoplasms” or “Endometrial Neoplasm” or “Neoplasm, Endometrial” or “Neoplasms, Endometrial” or “Endometrial Carcinoma” or “Carcinoma, Endometrial” or “Carcinomas, Endometrial” or “Endometrial Carcinomas” or “Endometrial Cancer” or “Cancer, Endometrial” or “Cancers, Endometrial” or “Endometrial Cancers” or “Endometrium Cancer” or “Cancer, Endometrium” or “Cancers, Endometrium” or “Cancer of the Endometrium” or “Carcinoma of Endometrium” or “Endometrium Carcinoma” or “Endometrium Carcinomas” or “Cancer of Endometrium” or “Endometrium Cancers”) 2. TS=(“Immune Checkpoint Inhibitors” or “Checkpoint Inhibitors, Immune” or “Immune Checkpoint Inhibitor” or “Checkpoint Inhibitor, Immune” or “Immune Checkpoint Blockers” or “Checkpoint Blockers, Immune” or “Immune Checkpoint Blockade” or “Checkpoint Blockade, Immune” or “Immune Checkpoint Inhibition” or “Checkpoint Inhibition, Immune” or “PD-L1 Inhibitors” or “PD L1 Inhibitors” or “PD-L1 Inhibitor” or “PD L1 Inhibitor” or “Programmed Death-Ligand 1 Inhibitors” or “Programmed Death Ligand 1 Inhibitors” or “PD-1-PD-L1 Blockade” or “Blockade, PD-1-PD-L1”or “PD 1 PD L1 Blockade” or “PD-1 Inhibitors” or “PD 1 Inhibitors” or “PD-1 Inhibitor” or “Inhibitor, PD-1”or “PD 1 Inhibitor” or “Programmed Cell Death Protein 1 Inhibitor” or “Programmed Cell Death Protein 1 Inhibitors” ) 3. TS=(“Durvalumab” or “MEDI4736” or “MEDI-4736” or “Imfinzi” or “Pembrolizumab” or “SCH-900475” or “lambrolizumab” or “MK-3475” or “Keytruda” or “Dostarlimab” or “Jemperli” or “dostarlimab-gxly” or “TSR-042” or “GSK4057190” or “Atezolizumab” or “anti-PDL1” or “immunoglobulin G1, anti-(human CD antigen CD274) (human monoclonal MDPL3280a heavy chain), disulfide with human monoclonal MDPL3280a kappa-chain, dimer” or “MPDL3280A” or “MPDL-3280A” or “Tecentriq” or “RG7446” or “RG-7446” or “Nivolumab” or “Opdivo” or “ONO-4538” or “ONO 4538” or “ONO4538” or “MDX-1106” or “MDX 1106” or “MDX1106” or “BMS-936558” or “BMS 936558” or “BMS936558” or “Avelumab” or “MSB-0010682” or “MSB0010682” or “bavencio” or “MSB0010718C” or “MSB-0010718C”) 4. #2 or #3 5. TI=(random* OR placebo* OR trial) and Preprint Citation Index (Exclude – Database) 6. AB=(random* OR placebo*) 7. #5 or #6 8. TS=(cohort studies* OR case-control studies* OR comparative study* OR risk factors* OR cohort* OR compar* OR group* OR "case control*" OR multivariat*) 9. #7 or #8 10. #1 and #4 and #9 |
| **Chochrane libarary** |
| **#1** "Endometrial Neoplasms"[Mesh]  **#2** "Endometrial Neoplasms"[Title/Abstract] OR "Endometrial Neoplasm"[Title/Abstract] OR "Neoplasm, Endometrial"[Title/Abstract] OR "Neoplasms, Endometrial"[Title/Abstract] OR "Endometrial Carcinoma"[Title/Abstract] OR "Carcinoma, Endometrial"[Title/Abstract] OR "Carcinomas, Endometrial"[Title/Abstract] OR "Endometrial Carcinomas"[Title/Abstract] OR "Endometrial Cancer"[Title/Abstract] OR "Cancer, Endometrial"[Title/Abstract] OR "Cancers, Endometrial"[Title/Abstract] OR "Endometrial Cancers"[Title/Abstract] OR "Endometrium Cancer"[Title/Abstract] OR "Cancer, Endometrium"[Title/Abstract] OR "Cancers, Endometrium"[Title/Abstract] OR "Cancer of the Endometrium"[Title/Abstract] OR "Carcinoma of Endometrium"[Title/Abstract] OR "Endometrium Carcinoma"[Title/Abstract] OR "Endometrium Carcinomas"[Title/Abstract] OR "Cancer of Endometrium"[Title/Abstract] OR "Endometrium Cancers"[Title/Abstract]  **#3** #1 OR #2  **#4** "Immune Checkpoint Inhibitors"[Mesh]  **#5** "Immune Checkpoint Inhibitors"[Title/Abstract] OR "Checkpoint Inhibitors, Immune"[Title/Abstract] OR "Immune Checkpoint Inhibitor"[Title/Abstract] OR "Checkpoint Inhibitor, Immune"[Title/Abstract] OR "Immune Checkpoint Blockers"[Title/Abstract] OR "Checkpoint Blockers, Immune"[Title/Abstract] OR "Immune Checkpoint Blockade"[Title/Abstract] OR "Checkpoint Blockade, Immune"[Title/Abstract] OR "Immune Checkpoint Inhibition"[Title/Abstract] OR "Checkpoint Inhibition, Immune"[Title/Abstract] OR "PD-L1 Inhibitors"[Title/Abstract] OR "PD L1 Inhibitors"[Title/Abstract] OR "PD-L1 Inhibitor"[Title/Abstract] OR "PD L1 Inhibitor"[Title/Abstract] OR "Programmed Death-Ligand 1 Inhibitors"[Title/Abstract] OR "Programmed Death Ligand 1 Inhibitors"[Title/Abstract] OR "PD-1-PD-L1 Blockade"[Title/Abstract] OR "Blockade, PD-1-PD-L1"or "PD 1 PD L1 Blockade"[Title/Abstract] OR "PD-1 Inhibitors"[Title/Abstract] OR "PD 1 Inhibitors"[Title/Abstract] OR "PD-1 Inhibitor"[Title/Abstract] OR "Inhibitor, PD-1"or "PD 1 Inhibitor"[Title/Abstract] OR "Programmed Cell Death Protein 1 Inhibitor"[Title/Abstract] OR "Programmed Cell Death Protein 1 Inhibitors"[Title/Abstract]  **#6** "Durvalumab"[Title/Abstract] OR "MEDI4736"[Title/Abstract] OR "MEDI-4736"[Title/Abstract] OR "Imfinzi"[Title/Abstract] OR "Pembrolizumab"[Title/Abstract] OR "SCH-900475"[Title/Abstract] OR "lambrolizumab"[Title/Abstract] OR "MK-3475"[Title/Abstract] OR "Keytruda"[Title/Abstract] OR "Dostarlimab"[Title/Abstract] OR "Jemperli"[Title/Abstract] OR "dostarlimab-gxly"[Title/Abstract] OR "TSR-042"[Title/Abstract] OR "GSK4057190"[Title/Abstract] OR "Atezolizumab"[Title/Abstract] OR "anti-PDL1"[Title/Abstract] OR "immunoglobulin G1, anti-(human CD antigen CD274) (human monoclonal MDPL3280a heavy chain), disulfide with human monoclonal MDPL3280a kappa-chain, dimer"[Title/Abstract] OR "MPDL3280A"[Title/Abstract] OR "MPDL-3280A"[Title/Abstract] OR "Tecentriq"[Title/Abstract] OR "RG7446"[Title/Abstract] OR "RG-7446"[Title/Abstract] OR "Nivolumab"[Title/Abstract] OR "Opdivo"[Title/Abstract] OR "ONO-4538"[Title/Abstract] OR "ONO 4538"[Title/Abstract] OR "ONO4538"[Title/Abstract] OR "MDX-1106"[Title/Abstract] OR "MDX 1106"[Title/Abstract] OR "MDX1106"[Title/Abstract] OR "BMS-936558"[Title/Abstract] OR "BMS 936558"[Title/Abstract] OR "BMS936558"[Title/Abstract] OR "Avelumab"[Title/Abstract] OR "MSB-0010682"[Title/Abstract] OR "MSB0010682"[Title/Abstract] OR "bavencio"[Title/Abstract] OR "MSB0010718C"[Title/Abstract] OR "MSB-0010718C"[Title/Abstract]  **#7** #4 or #5 or #6  **#8** #3 and #7  **#9** ("randomized controlled trial"[Publication Type] OR "controlled clinical trial"[Publication Type] OR "randomized"[Title/Abstract] OR "placebo"[Title/Abstract] OR "drug therapy"[Mesh Subheading] OR "randomly"[Title/Abstract] OR "trial"[Title/Abstract] OR "groups"[Title/Abstract]) NOT ("animals"[MeSH Terms] NOT "humans"[MeSH Terms]) or "clinical trials as topic"[mesh] OR "random allocation"[mesh] OR "double-blind method"[mesh] OR "single-blind method"[mesh] OR "clinical trial"[pt] OR "research design"[mesh:noexp] OR "comparative study"[pt] OR "evaluation studies"[pt] OR "follow-up studies"[mesh] OR "prospective studies"[mesh] OR "cross-over studies"[mesh] OR "clinical trial"[tw] OR ((singl*[tw] OR doubl*[tw] OR trebl*[tw]) AND (mask*[tw] OR blind*[tw])) OR placebo*[tw] OR random*[tw] OR "control"[tw] OR "controls"[tw] OR prospectiv*[tw] OR volunteer*[tw] or "cohort studies"[mesh] OR "case-control studies"[mesh] OR "comparative study"[pt] OR "risk factors"[mesh] OR "cohort"[tw] OR "compared"[tw] OR "groups"[tw] OR "case control"[tw] OR "multivariate"[tw]  **#10**  #8 and #9 |
| **Embase** |
| ('endometrium tumor'/exp OR 'endometrium tumor' OR 'endometrial neoplasia':ab,ti OR 'endometrial neoplasm':ab,ti OR 'endometrial neoplasms':ab,ti OR 'endometrial tumor':ab,ti OR 'endometrial tumorigenesis':ab,ti OR 'endometrial tumour':ab,ti OR 'endometrium neoplasm':ab,ti OR 'endometrium tumour':ab,ti OR 'neoplasia of the endometrium':ab,ti OR 'neoplasm of the endometrium':ab,ti OR 'neoplastic endometrial':ab,ti OR 'neoplastic endometrium':ab,ti OR 'tumor of the endometrium':ab,ti OR 'tumour of the endometrium':ab,ti OR 'endometrium tumor':ab,ti) AND ('imfinzi':ab,ti OR 'medi 4736':ab,ti OR 'medi4736':ab,ti OR 'durvalumab':ab,ti OR 'bcd 201':ab,ti OR 'bcd201':ab,ti OR 'keytruda':ab,ti OR 'lambrolizumab':ab,ti OR 'mk 3475':ab,ti OR 'mk3475':ab,ti OR 'pbp 2102':ab,ti OR 'pbp2102':ab,ti OR 'sch 900475':ab,ti OR 'sch900475':ab,ti OR 'xtrudane':ab,ti OR 'pembrolizumab':ab,ti OR 'anb 011':ab,ti OR 'anb011':ab,ti OR 'dostarlimab gxly':ab,ti OR 'dostarlimab-gxly':ab,ti OR 'gsk 4057190':ab,ti OR 'gsk4057190':ab,ti OR 'jemperli':ab,ti OR 'tsr 042':ab,ti OR 'tsr042':ab,ti OR 'wbp 285':ab,ti OR 'wbp285':ab,ti OR 'dostarlimab':ab,ti OR 'monoclonal antibody mpdl 3280a':ab,ti OR 'monoclonal antibody mpdl3280a':ab,ti OR 'mpdl 3280a':ab,ti OR 'mpdl3280a':ab,ti OR 'rg 7446':ab,ti OR 'rg7446':ab,ti OR 'ro 5541267':ab,ti OR 'ro5541267':ab,ti OR 'tecentriq':ab,ti OR 'tecntriq':ab,ti OR 'atezolizumab':ab,ti OR 'immune checkpoint inhibitor'/exp OR 'immune checkpoint blocker':ab,ti OR 'immune checkpoint inhibitors':ab,ti OR 'immune checkpoint inhibitor':ab,ti) AND (('controlled clinical trial'/exp OR random*:ti,ab OR placebo*:ti,ab OR trial:ti) AND [embase]/lim OR 'clinical article'/exp) |

**TableS2.** The risk of bias of randomized controlled trials.

| **Study** | **Randomization process** | **Deviations from intended interventions** | **Missing outcome data** | **Measurement of the outcome** | **Selection of reported result** | **Overall** |
| --- | --- | --- | --- | --- | --- | --- |
| Westin et al 2023, DUO-E | Low | Low | Low | Low | Low | Low |
| Eskander et al 2023, GY018 | Low | Low | Low | Low | Low | Low |
| Makker et al 2023, 309/KEYNOTE-775 | Some concerns | Low | Low | Low | Low | Some concerns |
| Mirza et al 2023, RUBY | Low | Low | Low | Low | Low | Low |
| N. Colombo et al 2023, AtTEnd | Low | Low | Low | Low | Low | Low |
| Pignata et al 2023, MITO END-3 | Low | Low | Low | Low | Low | Low |

| **Study** | **Representativeness of the exposed cohort** | **Selection**  **of the non-exposed cohort** | **Ascertainment of exposure** | **Outcome of interest not present at start of study** | **Comparability of cohorts** | **Assessment of outcome** | **follow-up long enough** | **Adequacy of follow up** | **Score** |
| --- | --- | --- | --- | --- | --- | --- | --- | --- | --- |
| Lheureux et al.  2022 | 1 | 1 | 1 | 1 | 2 | 0 | 1 | 1 | 8 |
| Madariaga et al.  2023 | 1 | 1 | 1 | 1 | 2 | 0 | 1 | 1 | 8 |
| Mathews et al.  2022 | 1 | 0 | 1 | 1 | 1 | 0 | 1 | 1 | 6 |
| Liao et al.  2022 | 1 | 1 | 1 | 1 | 0 | 0 | 1 | 1 | 6 |
| Cui et al.  2022 | 1 | 0 | 1 | 1 | 0 | 0 | 1 | 1 | 5 |

**TableS3.** The Newcastle-Ottawa Quality Assessment Scale of the included non-randomized controlled studies

The Newcastle-Ottawa Quality Assessment Scale provides a score from 0 to 9 stars. Studies with higher scores are considered to have better methodological quality. A score of 7 or more stars is considered high quality, 5 to 6 stars as medium quality, and fewer than 5 stars as low quality.
